# Smad4 regulates growth plate matrix production and chondrocyte polarity

**DOI:** 10.1242/bio.021436

**Published:** 2017-02-06

**Authors:** Amanda T. Whitaker, Ellora Berthet, Andrea Cantu, Diana J. Laird, Tamara Alliston

**Affiliations:** 1 Department of Orthopaedic Surgery, University of California San Francisco, San Francisco, CA 94143, USA; 2 Department of Orthopaedic Surgery, Nationwide Children's Hospital, Columbus, Ohio 43205, USA; 3 Department of Obstetrics, Gynecology & Reproductive Sciences, University of California San Francisco, San Francisco, CA 94143, USA; 4 Eli and Edythe Broad Center of Regeneration Medicine and Stem Cell Research, University of California San Francisco, San Francisco, CA 94143, USA; 5 Department of Bioengineering and Therapeutic Sciences, University of California San Francisco, San Francisco, CA 94143, USA; 6 Department of Otolaryngology – Head and Neck Surgery, University of California San Francisco, San Francisco, CA 94143, USA

**Keywords:** Smad4, Growth plate, Polarity, Skeletal dysplasia

## Abstract

Smad4 is an intracellular effector of the TGFβ family that has been implicated in Myhre syndrome, a skeletal dysplasia characterized by short stature, brachydactyly and stiff joints. The TGFβ pathway also plays a critical role in the development, organization and proliferation of the growth plate, although the exact mechanisms remain unclear. Skeletal phenotypes in Myhre syndrome overlap with processes regulated by the TGFβ pathway, including organization and proliferation of the growth plate and polarity of the chondrocyte. We used *in vitro* and *in vivo* models of Smad4 deficiency in chondrocytes to test the hypothesis that deregulated TGFβ signaling leads to aberrant extracellular matrix production and loss of chondrocyte polarity. Specifically, we evaluated growth plate chondrocyte polarity in tibiae of Col2-Cre^+/−^;Smad4^fl/fl^ mice and in chondrocyte pellet cultures. *In vitro* and *in vivo*, Smad4 deficiency decreased aggrecan expression and increased MMP13 expression. Smad4 deficiency disrupted the balance of cartilage matrix synthesis and degradation, even though the sequential expression of growth plate chondrocyte markers was intact. Chondrocytes in Smad4-deficient growth plates also showed evidence of polarity defects, with impaired proliferation and ability to undergo the characteristic changes in shape, size and orientation as they differentiated from resting to hypertrophic chondrocytes. Therefore, we show that Smad4 controls chondrocyte proliferation, orientation, and hypertrophy and is important in regulating the extracellular matrix composition of the growth plate.

## Abstract (precis)

**Summary:** Smad4 is a key regulator of extracellular matrix production and chondrocyte proliferation, shape and orientation in the growth plate. Smad4 dysregulation results in skeletal dysplasias, such as Myhre syndrome.

## INTRODUCTION

The physis, or growth plate, is comprised of precisely organized chondrocytes that confer longitudinal growth of the bone through interactions of multiple signaling pathways that cooperate to control chondrocyte shape, polarity, proliferation, differentiation, and apoptosis (Abad et al., 1999; Sasaki et al., 2001). Disruption of these cellular events, either by trauma or genetic mutation, leads to physeal defects that can result in skeletal deformities and abnormal limb growth. In one such example, dysregulation of Smad4, a common intracellular effector of all transforming growth factor β (TGFβ) family members, is responsible for human Myhre syndrome and characterized by short stature, brachydactyly, and joint stiffness (Caputo et al., 2012; Le Goff et al., 2012; Caputo et al., 2014). Likewise, in the physis of Smad4-deficient mice, the columnar organization is visibly disrupted (Zhang et al., 2005). These skeletal malformations demonstrate the importance of the TGFβ pathway in physeal development. However, the cellular basis of physeal defects in Smad4-deficient mice and skeletal deformities in Myhre syndrome remains unclear.

Longitudinal growth of the physis progresses through three distinct cellular zones with key physical and biochemical features: resting, proliferative, and hypertrophic. Resting zone chondrocytes are round, have an irregular organization, are small in size, and express collagen type II. Proliferating chondrocytes change shape into flattened ovoid disks organized into columns of cells that express type II collagen. These proliferating cells express high levels of cyclin D1, which drives the G1/S transition in mitosis. Proliferating chondrocytes exhibit polarized localization of organelles such as primary cilia (de Andrea et al., 2010; Belluoccio et al., 2010). Hypertrophic chondrocytes no longer proliferate, but generate longitudinal growth through a coordinated series of events that begins with longitudinal cellular hypertrophy. The hypertrophic chondrocytes ultimately undergo chondrocyte apoptosis or transdifferentiation, which gives rise to vascular invasion and replacement of calcified cartilage with new bone matrix (Park et al., 2015). These hypertrophic chondrocytes express collagen X and MMP13, a metalloproteinase that facilitates the conversion of cartilage to bone. These characteristic changes in chondrocyte shape, extracellular matrix synthesis, and orientation are tightly regulated in normal skeletal development and are disrupted in skeletal dysplasias (Wang et al., 2014; Minami et al., 2010).

Polarity is a cell-intrinsic mechanism that controls cell shape, adhesion, and organelle distribution and is critical for the control of cell division, migration, and paracrine signaling (Gao and Yang, 2013; Laird et al., 2011; Randall et al., 2012). Therefore, it is not surprising that cell polarity is instrumental in the coordinated, sequential progression of chondrocyte proliferation, shape and orientation throughout the growth plate (Gao and Yang, 2013; Abad et al., 1999; Randall et al., 2012). Relative to the mechanisms regulating growth plate chondrocyte differentiation, little is known about the role of polarity effectors in physeal development.

TGFβ family ligands share a common intracellular effector, Smad4, which heteromerizes with receptor-specific Smads 1, 2, 3, 5 and 8 to facilitate nuclear translocation and regulation of transcription. Smad4 is expressed in all four zones of the growth plate, and mice with mutations in Smad4 exhibit skeletal anomalies (Zhang et al., 2005; Sakou et al., 1999). Mice with a targeted ablation of Smad4 in the chondrocytes, Col2a1-Cre^+/−^;Smad4^fl/fl^, exhibit dwarfism and delayed ossification. The growth plates have broad, short chondrocyte columns and, although not the focus of the study, the figures in a previous study suggested randomly oriented proliferating chondrocytes that distort the normal direction of bone growth, but no quantification of their orientation was described. Limb deformities due to mutations in other components of TGFβ family signaling pathways also support the key role for Smad4 in the physis. The Smad4/TGFβ pathway has also been implicated in migration, adhesion, and cytoskeletal organization in other cell types (Sasaki et al., 2001; Derynck and Zhang, 2003; Piccolo et al., 2014); however, the ability of Smad4 to regulate cell polarity has not been examined in chondrocytes. Although many studies affirm the key role for Smad4 and TGFβ family signaling in chondrocytes, the mechanisms by which Smad4 maintains normal growth plate organization are not well-defined. Here, we find Smad4 regulates proteoglycan synthesis of the growth plate through aggrecan and MMP13 expression, alters proliferation, and changes outcomes of polarity including chondrocyte shape, size and orientation with the loss of Smad4.

## RESULTS

### Chondrocyte-intrinsic ablation of Smad4 disrupts the balance of extracellular matrix synthesis and degradation

**Fig. 1.**
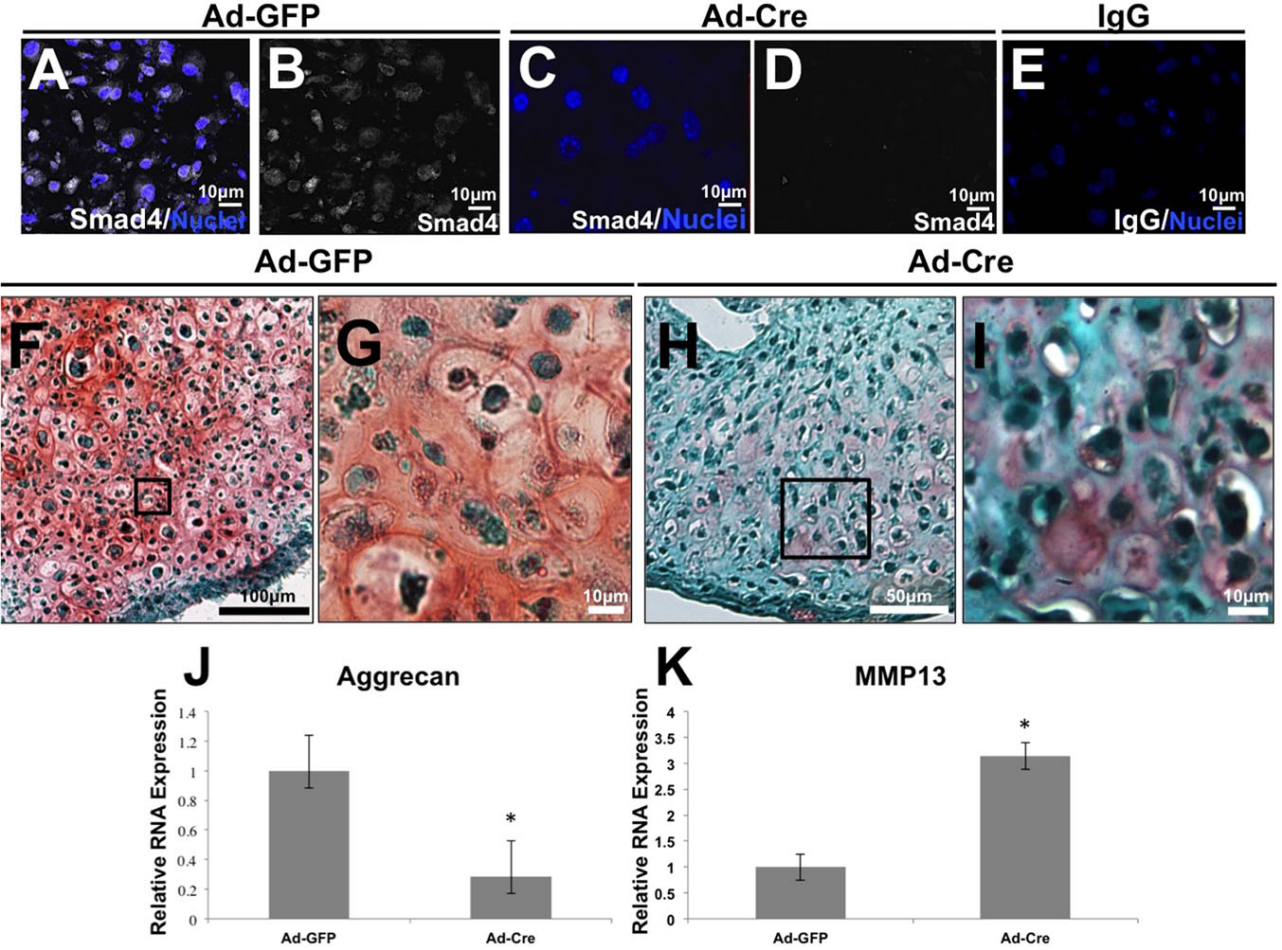
**Smad4 deficiency deregulates chondrocyte extracellular matrix homeostasis.** (A-E) Smad4 (white) is reduced in pellets of Smad4^fl/fl^ primary chondrocytes infected with adenovirus-Cre (Ad-Cre, C,D) relative to those infected with GFP control (Ad-GFP, A,B). Nuclei are counterstained with DAPI (blue). (F-I) Safranin O (pink) and Fast Green staining on primary chondrocyte pellet cultures demonstrate a Smad4-dependent loss of proteoglycan synthesis in the Ad-Cre Smad4^fl/fl^ chondrocyte pellets relative to Ad-GFP Smad4^fl/fl^ pellets. (J,K) Analysis of gene expression in primary Smad4^fl/fl^ chondrocytes infected with Ad-Cre or Ad-GFP demonstrate a Smad4-dependent decrease in aggrecan mRNA expression (J) and an increase in MMP13 mRNA expression (K). Expression was normalized to mRNA for ribosomal protein L19. *N*=3, *P*<0.05.

To evaluate the chondrocyte-intrinsic role of Smad4, excision was induced from primary chondrocytes of Smad4^fl/fl^ mice by adenoviral infection with Cre recombinase (Ad-Cre) *in vitro*. Loss of Smad4 expression was verified 2 days after addition of Ad-Cre (Fig. 1A-E). Following 21 days of pellet culture in chondrogenic conditions, Safranin O staining revealed decreased proteoglycan content in Smad4-deficient pellets relative to control pellets comprised of chondrocytes infected with adenovirus expressing green fluorescent protein (Ad-GFP) (Fig. 1F-I). Use of this pellet model allowed for discrimination of the cell-intrinsic role of Smad4 in chondrocytes from the potential role of Smad4 in the perichondrium, vasculature, or other tissues of the physis.

We examined mRNA expression of aggrecan and MMP13 to determine if the Smad4-dependent defect in extracellular matrix (ECM) composition corresponds to an imbalance in the expression of anabolic and catabolic genes, as it does in Smad3-deficient chondrocytes (Chen et al., 2012). We observed that transcript levels of aggrecan, the principal proteoglycan in cartilage, were decreased in primary chondrocyte cultures lacking Smad4 (Ad-Cre) relative to wild-type controls (Ad-GFP) (Fig. 1J). Conversely, primary chondrocytes lacking Smad4 showed elevated mRNA expression of matrix metallopeptidase 13 (MMP13), a protease that cleaves collagen II and aggrecan (Fig. 1K). Immunofluorescence indicates that Smad4-deficient primary chondrocyte cultures also express elevated levels of collagen X, which may suggest a trend toward a hypertrophic phenotype (Fig. S1). Therefore, Smad4 is required to maintain the balance between expression of aggrecan and MMP13, such that chondrocyte-intrinsic Smad4 deficiency favors matrix degradation with increased catabolic MMP13 and decreased anabolic aggrecan.

**Fig. 2.**
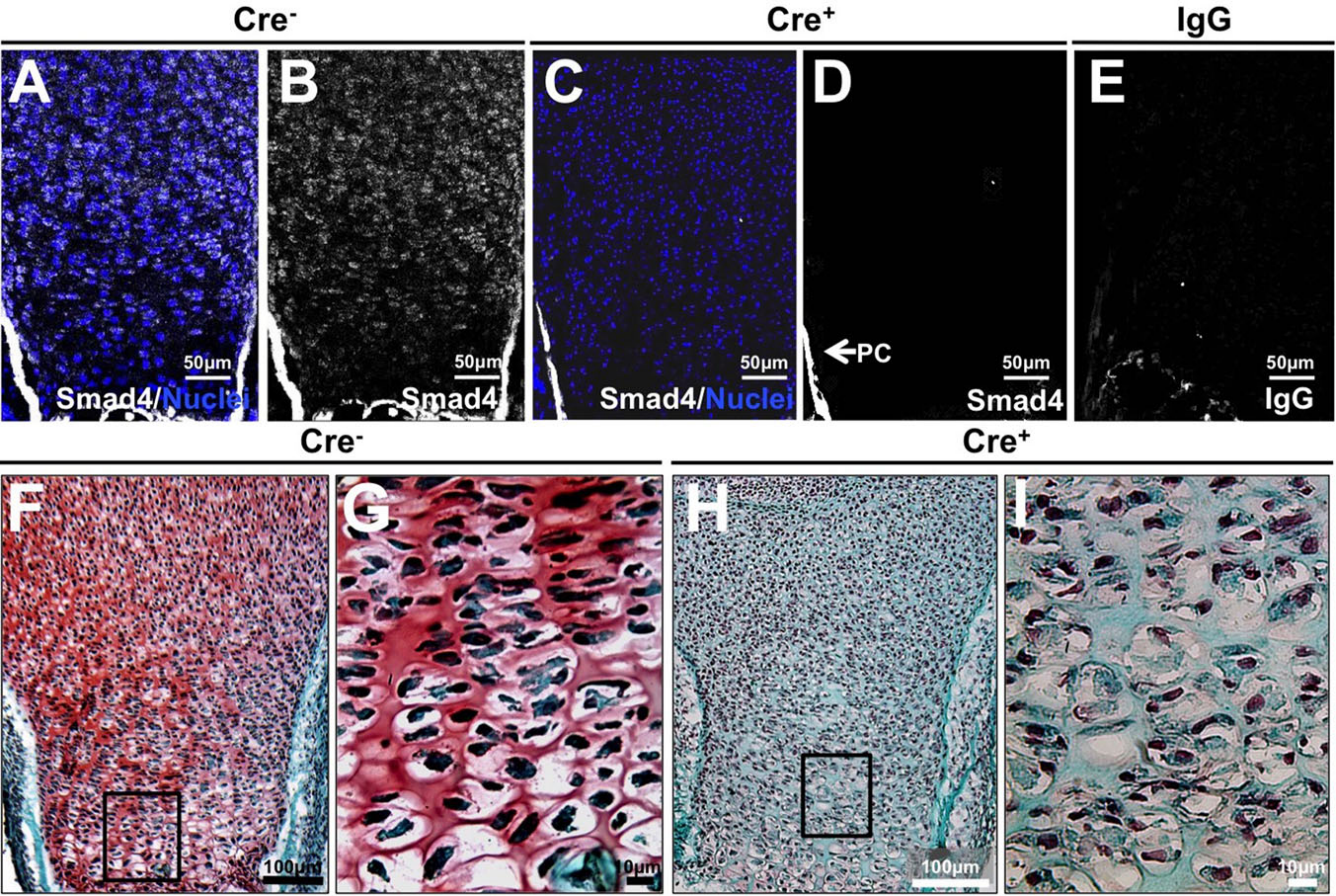
**Smad4-dependent reduction in growth plate proteoglycan *in vivo*.** (A-E) Immunofluorescence for Smad4 in E18.5 tibial growth plates demonstrate absence of Smad4 in the cartilage of the Col2-Cre^+/−^;Smad4^fl/fl^ (Cre^+^) mouse (nuclei: blue, Smad4: gray). (F-I) Safranin O (pink) and Fast Green staining on proximal tibial sections demonstrate a loss of proteoglycan synthesis in the Col2-Cre^+/−^;Smad4^fl/fl^ growth plate (Cre^+^; H,I) relative to the Cre^−^ control (F,G). *N*=3.

To examine the role of chondrocyte-intrinsic Smad4 in the intact growth plate, the floxed Smad4 allele was ablated in chondrocytes using a chondrocyte-specific Cre-recombinase under control of the Col2α1 promoter *in vivo* (Bardeesy et al., 2006; Chen et al., 2012). Immunofluorescence confirmed the loss of Smad4 protein expression in chondrocytes throughout the growth plate of embryonic day 18.5 Col2-Cre^+/−^;Smad4^fl/fl^ mice (Cre^+^), relative to Col2-Cre^−/−^;Smad4^fl/fl^ (Cre^−^) littermate controls (Fig. 2A-E). Smad4 expression is maintained in the perichondrium, consistent with the absence of Col2-Cre expression and activity in these cells (Ovchinnikov et al., 2000).

Owing to the dramatic proteoglycan loss in Smad4-deficient chondrocyte pellets *in vitro*, we examined the proteoglycan content of growth plates with a chondrocyte-intrinsic ablation of Smad4. Relative to the proteoglycan-rich growth plate of the wild-type Col2-Cre^−/−^;Smad4^fl/fl^ (Cre^−^) mice (Fig. 2F,G), the growth plates of Col2-Cre^+/−^;Smad4^fl/fl^ (Cre^+^) mice had a loss of proteoglycans (Fig. 2H,I). These findings are consistent with the chondrocyte pellet cultures **(**Fig. 1F-I**)**, confirming that chondrocyte-intrinsic Smad4 is necessary to maintain normal proteoglycan homeostasis in the growth plate.

**Fig. 3.**
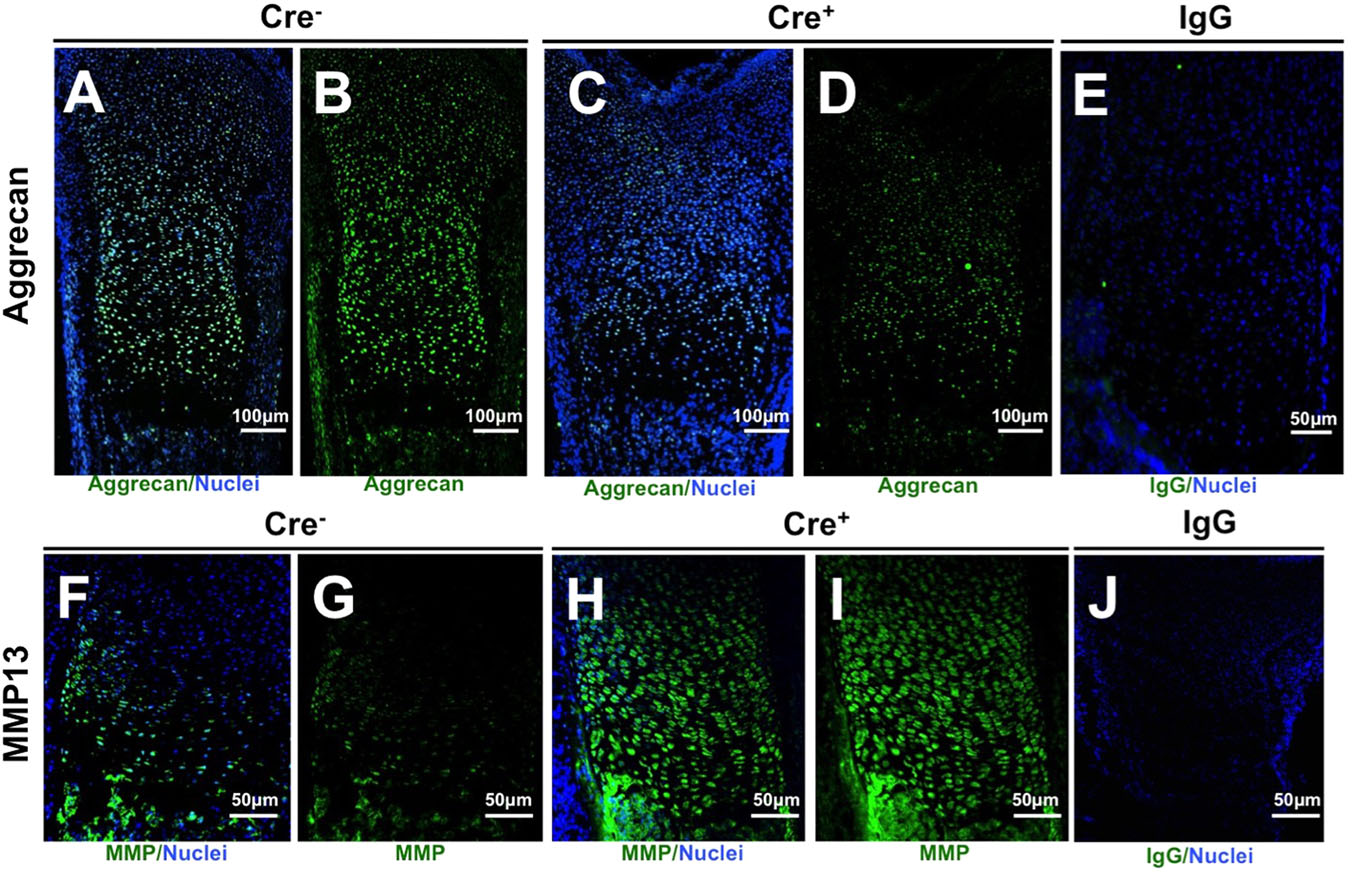
**Aggrecan is decreased and MMP13 increased in growth plates of Smad4-deficient mice.** (A-D) Immunofluorescence of tibial growth plates demonstrates decreased protein levels of aggrecan in the Col2-Cre^+/−^;Smad4^fl/fl^ (Cre^+^) growth plate (C,D) relative to the Cre^−^ growth plate (A,B); whereas MMP13 immunofluorescence of tibial growth plates demonstrates increased MMP13 protein in Col2-Cre^+/−^;Smad4^fl/fl^ (Cre^+^) growth plates (H, I), compared with Cre^−^ growth plates (F,G). IgG- negative controls for aggrecan (E) and MMP13 (J). *N*=3; A-D: nuclei, blue; Aggrecan, green; F-I: nuclei, blue; MMP13, green; E,J: nucleui, blue; IgG, green.

The defect in proteoglycan content in Smad4-deficient physeal cartilage was further evaluated using immunofluorescence for aggrecan and MMP13. Aggrecan expression was decreased in the Smad4-deficient growth plate compared with the wild-type growth plate (Fig. 3A-E). Thus, reduced aggrecan protein expression by chondrocyte-intrinsic ablation of Smad4 *in vivo* corresponds to the results in primary chondrocyte cultures (Fig. 1J). MMP13 expression was increased in physeal chondrocytes of Smad4-deficient mice, also consistent with the increased MMP13 mRNA expression in Smad4-deficient primary chondrocyte pellet cultures (Figs 3F-J and 1K). The increased MMP13 expression and content, decreased aggrecan expression and content, and decreased overall proteoglycan *in vitro* and *in vivo* indicate a key role for Smad4 in maintaining the composition of the growth plate extracellular matrix. This effect of Smad4 deficiency is chondrocyte-intrinsic, since it is displayed both *in vitro*, in chondrocyte cultures, and *in vivo* in growth plates.

### Smad4 is essential for growth plate chondrocyte proliferation, but not differentiation

**Fig. 4.**
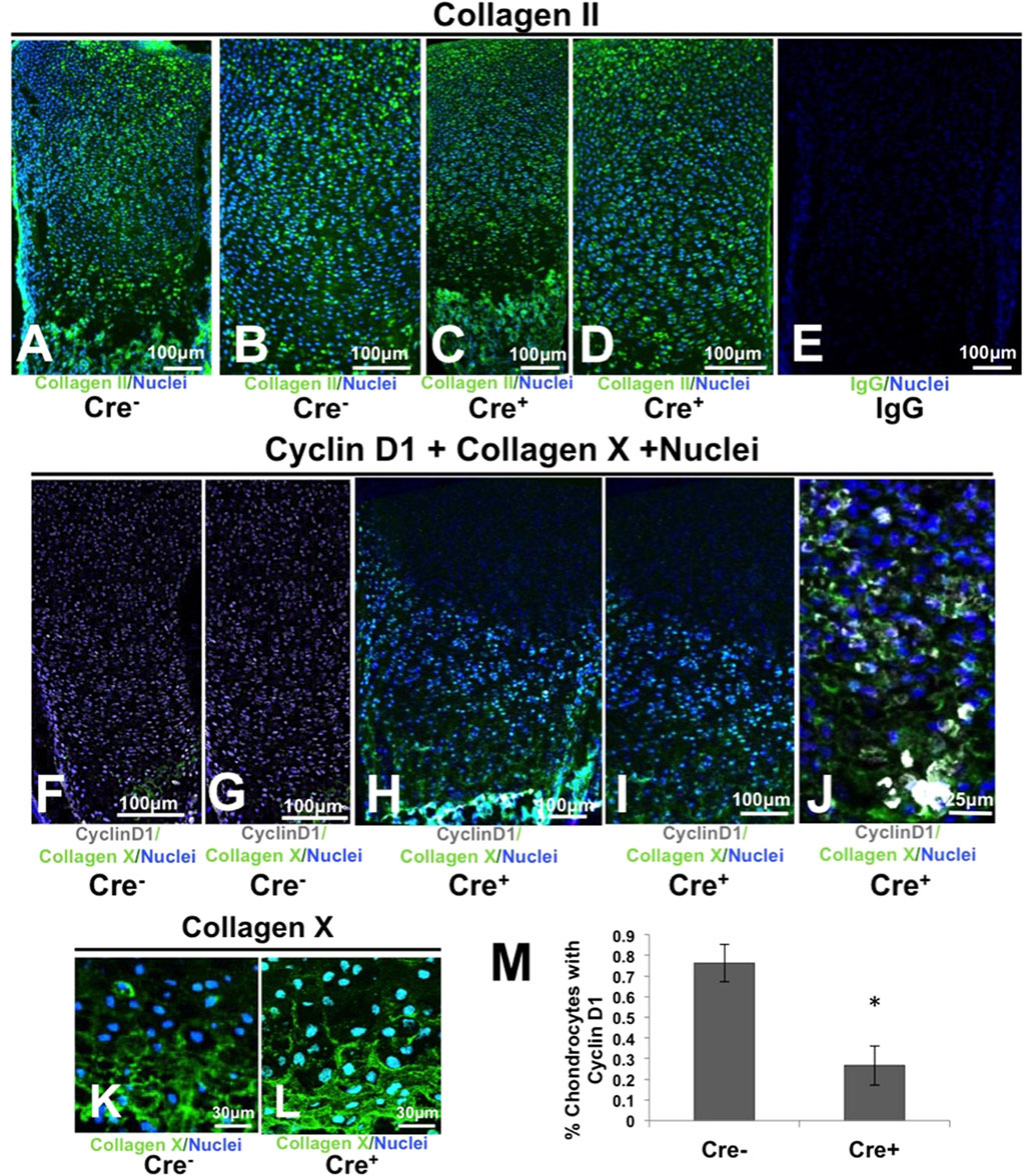
**Smad4 is essential for growth plate chondrocyte proliferation but is dispensable for differentiation.** (A-D) The level and distribution of collagen II and collagen X (K,L) are similar in wild-type (Cre^−^) and Smad4-deficient (Cre^+^) growth plates. Panels (B) and (D) are expansions of (A) and (C), respectively. (E) Negative IgG control for collagen II. (F-I) The frequency of Cyclin D1 expression is decreased in Smad4 deficient (Cre^+^) growth plates relative to wild-type Cre^−^ littermates. Panels (G) and (I) are expansions of (F) and (H), respectively. (J) In addition, Cre^+^ growth plates exhibit aberrant pockets of proliferative chondrocytes. (M) The number of proliferative cells in the Col2-Cre^+/−^;Smad4^fl/fl^ (Cre^+^) growth plate is reduced relative to Cre^−^ controls as demonstrated by a decrease in the percentage of cyclin D1-positive nuclei. Mean±s.d.; *P<0.01; Student's *t*-test, two tailed with equal variance. A-D: nuclei, blue; collagen II, green; F-L: nuclei, blue; collagen X, green.

To further examine the effect of Smad4 deficiency on physeal development, specific markers of growth plate zones were examined in Col2-Cre;Smad4^fl/fl^ mice by immunohistologic analysis. Collagen II is expressed by chondrocytes in the resting and proliferative zones, whereas collagen X is expressed by chondrocytes in the hypertrophic zone. The localization and level of collagen II and collagen X expression were similar between the wild-type and Smad4-deficient growth plates (Fig. 4A-E,K-L). This suggests that Smad4-deficient growth plate chondrocytes retain the capacity to produce the characteristic collagen matrix unique to the proliferative and hypertrophic zones of the physis.

Cyclin D1 is an established marker of cell proliferation that is functionally essential for the division of proliferating zone chondroctyes (Neven et al., 2007; Beier et al., 2001; Baldin et al., 1993). Cyclin D1 expression was decreased in Col2-Cre^+/−^;Smad4^fl/fl^ (Cre^+^) mice compared with wild-type mice (Cre^−^) (Fig. 4F-J**)**. Cre^+^ mice possessed 60% fewer CyclinD1-positive growth plate chondrocytes than wild-type littermates (Fig. 4M). The significant decrease in cyclin D1, but the normal expression of collagen II, suggests the cells have a defect in proliferation, but properly differentiate into proliferative zone chondrocytes. In addition, aberrant pockets of cyclin D1-positive cells were frequently found in the hypertrophic zones of the Cre^+^ growth plates but not in Cre^−^ wild-type mice (Fig. 4J). Collectively, these data suggest a Smad4-dependent defect in the regulation of chondrocyte proliferation, which confirms a previous study (Zhang et al., 2005).

### Smad4 deficiency changes chondrocyte cell shape, size and orientation

**Fig. 5.**
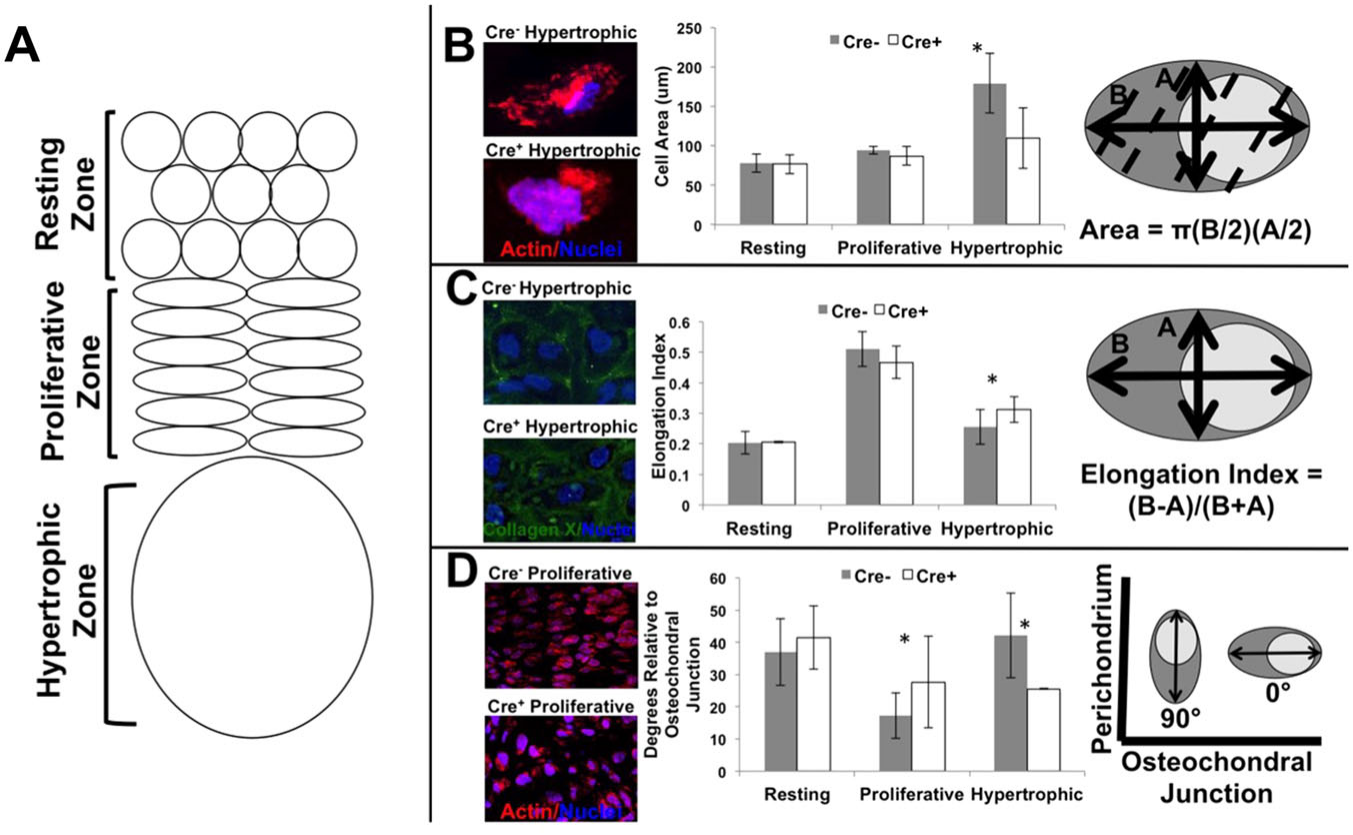
**Smad4 deficiency disrupts several markers of chondrocyte polarity including cell area, elongation index, and cellular orientation.** (A) Diagram of the cell shape and orientation of the resting, proliferative, and hypertrophic zones of the growth plate. (B) Chondrocyte hypertrophy is decreased by Smad4 deficiency (Cre^+^) as expressed by a reduced cell area in the hypertrophic zone with diagram of the cell area measurement. (C) Col2-Cre^+/−^;Smad^fl/fl^ (Cre^+^) hypertrophic chondrocytes are slightly but significantly more elongated than the wild type (Cre^−^) chondrocytes with diagram of the elongation index measurement. (D) The long axis relative to the osteochondral junction of Col2a1-Cre^+/−^;Smad4^fl/fl^ (Cre^+^) chondrocytes is reversed with a vertical orientation in the proliferative zone and a horizontal orientation in the hypertrophic zone with diagram of the long axis orientation measurement. In B-D, significance was determined by *P<0.05 using a two-tailed Student's *t*-test; mean±s.d.

Chondrocytes adopt a distinctive cell shape and orientation at each stage of their differentiation in the growth plate (Buckwalter et al., 1985). In the resting zone, chondrocytes are round. In the proliferative zone, chondrocytes are elliptical, or disk shaped, with their long axis perpendicular to the direction of growth (Li and Dudley, 2009). Hypertrophic chondrocytes are large and elongated in the direction of growth, with their proximal to distal length greater than half of the width (Fig. 5A). As the chondrocytes mature, they expand in area, especially in the hypertrophic zone. Unlike the classical appearance of the growth plate in their wild-type littermates (Col2-Cre^−/−^;Smad4^fl/fl^), these hallmarks were less apparent in the growth plate chondrocytes of the Col2-Cre^+/−^;Smad4^fl/fl^ (Cre+) mice (Fig. 2G,I).

Since cell size, shape, and orientation depend on normal regulation of cell polarity, we hypothesized that polarity control was impaired in Smad4-deficient chondrocytes. To test this hypothesis, we used immunofluorescence and confocal microscopic analysis to visualize and quantify polarity outcomes in growth plate chondrocytes from Cre^+^ mice and their Cre^−^ littermates (Fig. 5B-D). Actin visualized with phalloidin allowed detection of cell boundaries, from which the cell area was quantified. Hypertrophic chondroctyes from wild-type (Cre^−^) growth plates undergo a twofold increase in cell area. In contrast, Smad4-deficient Col2-Cre^+/−^;Smad4^fl/fl^ (Cre^+^) chondrocytes do not undergo hypertrophy, but maintain a small size similar to that of the resting and proliferative chondrocyte (Fig. 5B).

Another measure of cell polarity, the elongation index, was used to quantify cell shape (Laird et al., 2011; Randall et al., 2012). Elongation indices closer to 0 describe more round (or less elongated) cells, whereas indicies approach 1 for cells more elliptical in shape, or more elongated. In the wild-type (Cre^−^) and Smad4-deficient Col2-Cre^+/−^;Smad4^fl/fl^ (Cre^+^) growth plates, the resting zone chondrocytes were round with an elongation index of 0.2 (Fig. 5C). Wild-type (Cre^−^) and Smad4-deficient Col2-Cre^+/−^;Smad4^fl/fl^ (Cre^+^) chondrocytes become more elliptical as they enter the proliferative zone; however, Smad4-deficient chondrocytes showed a significant decrease in their ability to return to a rounded shape during the transition to the hypertrophic zone.

Smad4-deficient (Cre^+^) growth plate chondrocytes also demonstrated a diminished capacity to reorient their long axis with respect to the direction of growth, another element of polarity (Fig. 5D). Whereas wild-type proliferating chondrocytes undergo an average 30° shift in axis orientation relative to the osteochondral junction from the resting to proliferative zone, the long axis of Smad4-deficient (Cre^+^) proliferative chondrocytes shifted less than half as much (12°) during the same transition. Likewise, wild-type (Cre^−^) hypertrophic chondrocytes reoriented again with a 30° change in axis orientation relative to the osteochondral junction from the proliferative to the hypertrophic zone. This change was absent in Smad4-deficient hypertrophic chondrocytes (Fig. 5D). The change in long axis orientation is independent of perichondreal influences, as the perichondrium contains Smad4. Thus, Smad4 is required autonomously in chondrocytes for control of cellular shape and orientation in the growth plate, suggesting that molecular regulators of cellular polarity are disrupted in Smad4-deficient Col2-Cre^+/−^;Smad4^fl/fl^ (Cre^+^) chondrocytes.

Organelle localization within a cell also is a marker for polarity (Laird et al., 2011; de Andrea et al., 2010). In an effort to explore mechanisms underlying the Smad4-dependent differences in growth plate chondrocytes, we used immunofluorescence for several established organelle marker proteins to examine the localization of the Golgi, centrosomes, and primary cilia. The Golgi had a tendency to be more vertically oriented in Smad4-deficient proliferative chondrocytes (Fig. S2). We did not detect any changes in localization of primary cilia or centrosomes. Intracellular effectors of polarity pathways, such as Ror2, JNK, and β-catenin may also be affected by Smad4 dysfunction. Although we did not observe Smad4-dependent differences in the localization of the non-canonical Wnt receptor Ror2, JNK and β-catenin localization were altered in a Smad4-dependent manner (Figs S3 and S4). Additional studies are needed to explore the functional relevance of these Smad4-dependent changes in organelle and signaling protein localization in chondrocytes and during growth plate development.

## DISCUSSION

Precise networks of molecular and cellular events orchestrate growth plate organization and development. Among these critical cellular processes is the regulation of chondrocyte polarity, which is essential for the development of bones with the correct size and shape (de Andrea et al., 2010; Ahrens and Dudley, 2011). Our studies reveal a novel role for Smad4 in physeal matrix production and chondrocyte polarity and proliferation. Growth plates from mice with a chondrocyte-intrinsic ablation of Smad4 have severe deregulation of proteoglycan levels in spite of normal expression of growth plate chondrocyte differentiation markers. Smad4 facilitates the dramatic cellular changes that are apparent as cells differentiate from resting to hypertrophic chondrocytes. Chondrocytes of Smad4-deficient growth plates are limited in their ability to undergo characteristic changes in cell size, shape, or orientation, which are markers of polarity.

Smad4 deficiency changes the proteoglycan composition of the growth plate by altering the balance between extracellular matrix anabolism and catabolism. Proteoglycan content is reduced in the Smad4-deficient growth plate due to decreased aggrecan expression and increased MMP13 expression. The shift towards matrix catabolism by Smad4-deficient chondrocytes is apparent in primary chondrocyte monolayer, pellet cultures, and in the growth plate (Fig. 1F-I). Disorganized extracellular matrix in Smad4-deficient skin fibroblasts has also been noted (Piccolo et al., 2014). Chondrocyte-intrinsic Smad4 deficiency alters the function of the other receptor-activated Smads, such as the BMP-responsive Smads 1, 5 and 8, which also participate in endochondral ossification (Keller et al., 2011). This role of Smad4 has been suggested, as Smad4-coupled Smad2/3 increases aggrecan expression and inhibits MMP13 (Yamagata et al., 2012; Wang et al., 2014; Kawamura et al., 2012; Chen et al., 2012). Therefore, Smad4 plays a cell-intrinsic role in the regulation of proteoglycan homeostasis in the growth plate extracellular matrix.

The effect of Smad4-deficiency on chondrocyte polarity has significant implications for endochondral ossification and skeletal morphogenesis. Polarity pathways are highly conserved mechanisms that control cytoskeletal organization and subcellular organelle localization to facilitate cellular events from proliferation to migration. Throughout the animal kingdom, deregulation of polarity impairs cell proliferation and cell orientation, size, and shape. These defects are apparent in Smad4-deficient growth plate chondrocytes (Fig. 5). Previous work in a different Smad4-deficient mouse system described defective chondrocyte proliferation in mice with short, wide growth plates (Salazar et al., 2013; Zhang et al., 2005). Likewise, we find that chondrocyte-intrinsic Smad4 ablation results in decreased chondrocyte proliferation and a smaller proliferative zone, even when perichondrial Smad4 is intact (Fig. 4F-I). We also observed defects in other polarity-dependent processes, including the control of cell orientation, size, and shape. Smad4-deficient chondrocytes fail to change the orientation of their long axis as they transition from the proliferative zone to the hypertrophic zone (Fig. 5D). Chondrocyte size was reduced in the hypertrophic zone of the Smad4-deficient growth plate, even though *in vivo* chondrocytes expressed normal levels of the differentiation marker collagen X (Fig. 5B, Fig. 4K,L). Finally, unlike wild-type cells, Smad4-deficient hypertrophic chondrocytes fail to adopt a more rounded shape as they transition from flattened proliferative chondrocytes to stout hypertrophic chondrocytes (Fig. 5D). Accordingly, we observed a change in the Golgi body localization and the direction of chondrocyte division (Fig. S2). Together with other studies, the current study highlights the need for additional investigation into the mechanisms of Smad4 crosstalk with known polarity effectors, such as the non-canonical Wnt pathway (Romereim et al., 2014). Although the localization of Ror2 and primary cilia did not show conclusive Smad4-dependent differences, crosstalk with the β-catenin/WNT pathway and regulation of JNK may be one mechanism by which Smad4 regulates chondrocyte matrix and polarity through cell shape, size, and orientation. Because these polarity defects impact growth plate chondrocyte proliferation, orientation, size, and shape, all of which can impact skeletal morphogenesis, they likely contribute to skeletal dysplasias similar to Myhre syndrome.

The extent to which the deregulation of Smad4 growth plate chondrocyte polarity is cell-intrinsic or secondary to the deregulation of proteoglycans remains a critical area for future study. The alterations observed in chondrocyte shape and orientation, as well as organization, could be due to alterations in the proteoglycan content of the extracellular matrix. Extracellular matrix composition can change cell adhesion and signaling. Others have shown that cellular adhesions to extracellular matrix can influence chondrocyte cell shape, which in turn can impact planes of division and cell orientation (Romereim et al., 2014). Several aspects of the aggrecan-deficient growth plate resemble those observed in Smad4-deficient growth plates, including skeletal dysplasia, loss of aggrecan expression, reduced chondrocyte proliferation, and deregulation of chondrocyte shape and organization (Lauing et al., 2014). This is consistent with the pivotal role of aggrecan and other proteoglycans in regulating signaling through growth factors that depend on Smad4. Therefore, whether through changes in adhesion, growth factor signaling, or other mechanisms, our findings support the hypothesis that extracellular matrix composition could contribute to the apparent differences in cell shape and orientation.

Myhre syndrome is a skeletal dysplasia characterized by short stature, brachydactyly, and joint stiffness, with laryngotracheal stenosis and restrictive respiratory insufficiency leading to increased mortality as the result of Smad4 dysregulation (Caputo et al., 2012; Le Goff et al., 2012, 2014; Caputo et al., 2014). The defects we observed in Smad4-deficient chondrocyte proliferation, size, orientation, and shape, coupled with abnormal proteoglycan content, could contribute to the pathology of Myhre syndrome, most notably the joint stiffness, bracydactyly, restricted thoracic development, and short stature. Skeletal dysplasia due to Smad4 deficiency resembles other skeletal dysplasias resulting from mutations in polarity pathways, such as the Ror2/Wnt5a PCP pathway with Robinow syndrome and brachydactyly B2 (Afzal et al., 2000; Wang et al., 2010). The extent to which these phenotypic and cellular changes are accompanied by molecular crosstalk between polarity pathways and Smad4/TGFβ pathways remains to be determined. Understanding these molecular mechanisms may present new opportunities for therapeutic intervention for individuals with these syndromes.

## MATERIALS AND METHODS

### Col2-Cre and Smad4^fl/fl^ mice

Col2-Cre mice express Cre recombinase under the control of a 3 kb segment of the collagen II promoter. The excision of the Smad4 floxed loci occurs specifically in the growth plate chondrocytes and not in the perichondrium (Ovchinnikov et al., 2000). Smad4^fl/fl^ mice were generously donated by Dr D. Hanahan (EPFL, Lausanne, Switzerland; Bardeesy et al., 2006). PCR genotyping was performed on DNA isolated from tail biopsies using primers for the floxed and recombined Smad4 alleles (Bardeesy et al., 2006). The care and use of the mice was approved by the Institutional Animal Care and Use Committee at the University of California, San Francisco, USA. Proper euthanasia techniques were performed according to these standards.

### Replicates and statistical analysis

All figures are representative of multiple technical replicates of greater than or equal to three mice. At least five sections of each mouse growth plate were analyzed in each experiment. For the pellet cultures, at least three pellets with five technical replicates each were examined for each experiment. For cell count analysis of Cyclin D1, over 2000 cells were assessed in both wild-type (Cre^−^) and Smad4-deficient Col2-Cre^+/−^;Smad4^fl/fl^ (Cre^+^) chondrocytes in the collagen II producing zones, excluding those in the collagen X zones. Over 100 cells from at least three mice in each of the three zones for both wild-type (Cre^−^) and Smad4-deficient Col2-Cre^+/−^;Smad4^fl/fl^ (Cre^+^) growth plates were measured for area, elongation index, and degrees relative to the osteochondral junction. Student's *t*-test, two tailed, with equal variance was used for probability calculations to determine significance.

### Primary chondrocyte isolation

Primary chondrocytes were harvested from Smad4^fl/fl^ pups at ages post-natal day 2-4 by dissecting the cartilaginous bone ends from the proximal tibia, distal femur, proximal femur and proximal humerus. Chondrocytes were isolated from cartilaginous anlage by sterile incubation for 30 min shaking at 37°C in digestion media [Dulbeco's modification of eagle's medium, 45 mM sodium bicarbonate, 20 mM HEPES (pH 7.4), 0.01% Fungizone, 0.15 mg/ml gentamycin, 3 mg/ml collagenase D (Sigma)] as described (Rodriguez et al., 2005). Adenovirus with GFP/Cre-recombinase or GFP were added to the chondrocytes at 400 pfu/cell. Analysis of GFP expressing cells 12 h after infection verified that this protocol yields a 90% infection rate (Suter et al., 2006). All experiments used cells at third passage (after harvest digestion, infection and appropriate media change) at day 2 after initial harvest.

### Chondrocyte pellet culture

Primary chondrocyte pellets were produced from Smad4^fl/fl^ primary chondrocytes following infection with either adenoviral GFP or adenoviral GFP/Cre-recombinase. Pellets were produced by centrifugation of 500,000 cells at 57 ***g*** for 5 min at 4°C. The pellets were grown in chondrogenic media (Dulbeco's modification of eagle's medium, 100 µg/ml penicillin, 100 µ/ml streptomycin, and 10% by volume FBS) at 37°C for 21 days in 15 ml tubes prior to embedding for frozen sections.

### Frozen sections

Tibias dissected from embryonic day 18.5 Col2-Cre^+/−^;Smad4^fl/fl^ and Col2-Cre^−/−^;Smad4^fl/−^ mice were fixed in 4% paraformaldehyde (PFA) in PBS (Fink et al., 2003). Tibias were incubated in 5 mg/ml sodium borohydride for 2×30 min intervals to facilitate antigen retrieval prior to decalcification overnight in 19% EDTA (Ahrens and Dudley, 2011). Decalcification was confirmed radiographically. Tibias were then placed in 30% sucrose and 0.1 M potassium phosphate (pH 7.4) at 4°C overnight. The tibias were transferred to optimal cryogenic temperature (OCT) solution and frozen on dry ice. The blocks were sectioned in 9 µm sections and stored at −20°C.

### Immunofluorescence

Frozen sections of tibias and primary chondrocyte pellets were warmed to room temperature, washed with PBS, and fixed with 100% methanol or PFA. The sections were washed with PBS, 0.25% hyaluronidase prior to blocking with 10% heat-inactivated newborn calf serum in 0.1% tween in PBS. The following antibodies were used in the stated dilution: mouse anti-Smad4 (1:50, Santa-Cruz Biotechnology, B-8), mouse anti-cyclinD1 (1:50, Santa-Cruz Biotechnology 8396), rabbit anti-collagen X (1:100, Abcam 58632), rabbit anti-collagen II (1:100, Abcam), rabbit anti-Aggrecan (1:100, Developmental Studies Hybridoma Bank, 12/21/1-C-6), and anti-mouse MMP13 (1:100, Millipore, AB8120). Sections were incubated with rabbit IgG (1:100) or mouse IgG (1:100) as negative controls. All primary antibodies were incubated at 4°C overnight, whereas secondary antibodies (1:200) were incubated at room temperature for 1 h.

### Microscopy

Images were obtained using a Leica SP5 Laser Scanning Confocal microscope with 63× oil immersion, 40× oil immersion, 20× oil immersion and 10× air immersion objectives and 405, 488, 543, 594 and 633 nm lasers. Specific microscope settings were as follows: xy at 514×514, frame average of 3, and z-stacks at 0.5 µm. The z-stacks were then combined and saved as a PDF for cell shape and orientation analysis with ImageJ (NIH). The periosteum, joint surface, and osteochondral junction were used as landmarks to determine the growth plate orientation.

### Gene expression

RNA was isolated from primary chondrocyte pellets using the Purelink RNA mini Kit (Invitrogen). Primers for aggrecan and MMP13 have been described (Chen et al., 2012). The expression of each gene was normalized to the expression of L19 using the comparative CT method (Schmittgen and Livak, 2008). Amplification was verified by melting curve. Results were detected based on amplicon binding of SYBR Green (Bio-Rad) using the CFX96 Real-Time PCR Detection System (Bio-Rad).

### Cell polarity measurements

Using the ImageJ software, at 63× magnification, the image scale was calibrated. As shown in Fig. 5B, the longest axis of the cell was measured (line marked ‘B’) and a line perpendicular to the long axis was placed at the widest part of the cell (line marked ‘A’). The elongation index was calculated using the long axis minus the short axis divided by the sum (Laird et al., 2011). The long axis angulation was measured relative to the osteochondral junction, where 0° is parallel and 90° is perpendicular. Area of the cell was calculated using area of an ellipse equation [π(Long axis/2)(short axis/2)], and confirmed for accuracy by measuring the area using ImageJ software. To visualize cell shape and polarity, actin was visualized with phalloidin stain (Invitrogen) while nuclei were stained with DAPI (Vector Laboratories).
